# The high alpine bee fauna (Hymenoptera: Apoidea) of the Zillertal Alps, Austria

**DOI:** 10.3897/BDJ.2.e1115

**Published:** 2014-06-23

**Authors:** Silas Bossert

**Affiliations:** † Department of Integrative Zoology, University of Vienna, Vienna, Austria

**Keywords:** Tyrol, Zemmgrund Area, faunistic records, flower visits, *Bombus lucorum* complex, *Nomada glabella*

## Abstract

Bees from the Zemmgrund area in the Zillertal Alps (Austria, Tyrol) were collected and determined to investigate the species composition of the area. A total of 61 specimens were collected over a two year period; they represent 24 species from 8 genera. Building on these records, the first commented checklist for the area is presented, with notes on habitats and visited flowers.

## Introduction

Faunistic research on bees (Hymenoptera: Apoidea) in Tyrol enjoys a long tradition. Its foundations were laid by the great works of Dalla Torre (e.g. Dalla Torre 1873, Dalla Torre 1877a, Dalla Torre 1877b, Dalla Torre 1879, Dalla Torre 1882) and Schletterer (1887) that are related to the former County of Tyrol. Numerous complementary studies followed by a large number of authors concerning different parts of the region, for example the Ötztal Alps (Schedl 1982), Lower Inn Valley (Schuler 1982), Tiroler Mittelland (Stöckl 1996), Upper Inn Valley (Stöckl 1998), tyrolean Lech area (Schmid-Egger 2011) and Silvretta Alps together with Kleinwalsertal in Vorarlberg (Kuhlmann and Tumbrinck 1996). Bellmann and Hellrigl (1996) assembled a species list for South Tyrol which has been updated several times (Hellrigl 2006, Hellrigl and Franke 2004, Hellrigl 2003). Furthermore, Kopf (2008) provided an excellent treatise on the bees of the Schlern region. Overviews for different taxonomic groups of bees have been conducted by Gusenleitner (1985) regarding *Andrena* in Northern Tyrol and records of Halictidae from Northern Tyrol were prepared by Ebmer (1988). Stöckl (2000) worked on the Megachilinae in North and South Tyrol and Neumayer and Kofler (2005) evaluated the bumblebee fauna of eastern Tyrol. Additional faunistic data was assembled within the framework of the “GEO-Tage der Artenvielfalt” in Tyrol in 2005, 2006, 2009 and 2011. Aside from the data in Kopf et al. (2010), records for the Zillertal Alps are very rare, and for the Zemmgrund area only a list of bumblebee species for a small transect study (Penninger 2008) could be found in the literature. The aim of this study is to help close this knowledge gap and contribute the first extensive faunistic data set concerning the high alpine bee fauna of the Zillertal Alps.

## Materials and methods

Collections were conducted near the Berliner Hütte in three periods: July 4-10, 2012, July 3-9, 2013 and August 6-10, 2013. The focus within the area was on bees around and above the Berliner Hütte which is located on 2042 m above sea level. All sighted wild bees were collected manually and were transfered into an ethyl acetate killing jar. The majority of specimens were collected between an altitudinal range of 1850 m to 2400 m a.s.l. Four specimens were collected at lower altitudes as accidental findings during ascent and descent. GPS coordinates and altitudes were logged. The habitat of each collection site was categorized. Determinations were conducted using the identification keys of Amiet (Amiet 1996, Amiet et al. 1999, Amiet et al. 2001, Amiet et al. 2004, Amiet et al. 2007, Amiet et al. 2010), Dathe (Dathe 1977, Dathe 1980), Ebmer (Ebmer 1969, Ebmer 1971, Ebmer 1984), Gokcezade et al. (2010), Mauss (1994), Schmid-Egger and Scheuchl (1997) and Scheuchl (Scheuchl 1995, Scheuchl 2006). Critical specimens were sent to experts for examination: the author is indebted to Fritz Gusenleitner for helping with the specimens belonging to the genus *Andrena* and Maximilian Schwarz for specimens of *Nomada*. The suprageneric classification follows Michener (2007). All specimens are kept in the collection of the author. If the specimens were collected on flowers, the respective plant species was recorded. The plant species were determined using Rothmaler (2009) and Fischer et al. (2008). A list of the recorded plant species in the supplementary material (Suppl. material 1) provides further informations about the bee species visiting the respective flowers.

One collected specimen belongs to a cryptic bumblebee species group, the so-called *Bombus lucorum* complex. The status of three distinct species within the complex is widely accepted today (Bertsch et al. 2004, Bertsch et al. 2005, Murray et al. 2008, Bertsch 2009, Carolan et al. 2012, Williams et al. 2012). In contrast, there is heavy doubt about the species identification based on morphology and there are implications that the species might be morphologically indistinguishable (Williams 2000, Waters et al. 2011, Carolan et al. 2012). Therefore, a partial sequence of the mitochondrial COI gene from the specimen was ascertained to ensure the morphological determination. The specimen was stored in pure ethanol and a single crushed midleg was used for the analysis. DNA was extracted using a Proteinase K digestion prior to a phenol-chloroform protocol (Sambrook et al. 1989). The so-called “Folmer region” was amplified with Polymerase chain reactions (PCR) using the primers LCO1490 and HC02198 (Folmer et al. 1994). The remaining PCR components were provided using the DreamTaq™ PCR Mastermix (2x) (Thermo Fisher Scientific Inc., Waltham, MA, USA) and the amplification profile was conducted following the manufacturer´s protocol. The product was purified using the GeneJET™ PCR Purification Kit (Thermo Fisher Scientific Inc., Waltham, MA, USA) and sequencing was carried out by the VBC-Biotech Service GmbH (Vienna, Austria). The obtained sequence was checked manually using BioEdit 7.2.5 (Hall 1999). A BLAST search (Altschul et al. 1990), as implemented in GenBank, was conducted to estimate the query cover and identity to other sequences deposited in the databank.

**Figure 1.**
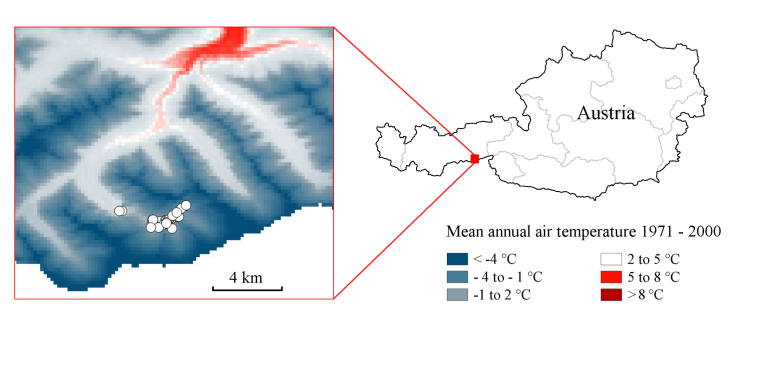
A temperature-based climate map of the study area and its localization in Austria. The map shows the mean annual air temperature for the period 1971 – 2000 with linearized color interpolation and is based on the data of Hiebl et al. (2011). The white circles indicate the collection localities. The mean annual air temperature of these localities, based on the years 1971 – 2000, range from -0.7 to 3.7 °C.

The climate map of the study area (Fig. 1) is based on the recently updated Austrian digital climate atlas from 1971-2000 (Hiebl et al. 2011). Given the importance of dependable data concerning the altitudinal climate changes, the high resolution Austrian climate maps for 1971-2000 consider altitudinal changes by a digital elevation model. The GIS grids were kindly provided by Alexander Orlik from the *Zentralanstalt für Meteorologie und Geodynamik* (ZAMG) and handled with QGIS 2.2 (QGIS Development Team 2014).

The microscope images were created using a SMZ25 stereomicroscope and a DS-Ri1 U3 microscope camera (Nikon Corp., Tokyo, Japan).

### Study area

**Figure 2.**
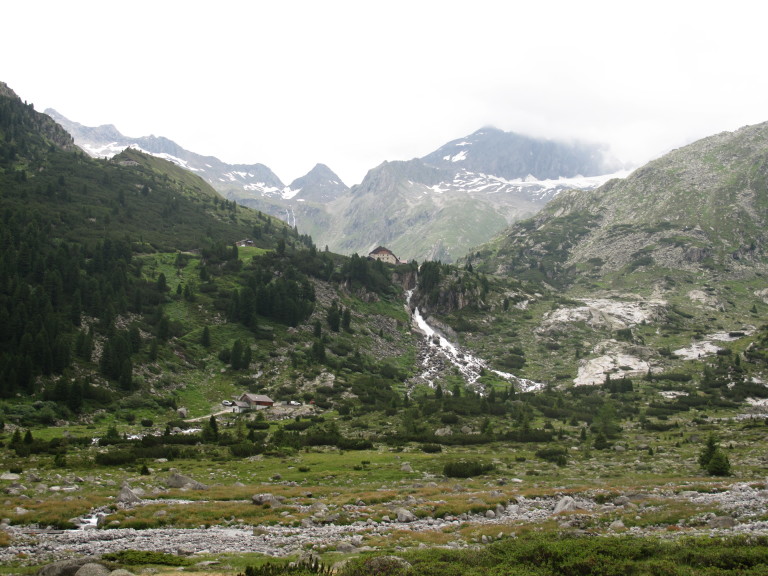
A photograph of the small scaled habitats that are characteristic for study area: A part of a Swiss pine forest (left side), aggregations of mountain pines (in the middle of the picture), an alpine pasture (in the foreground) and alpine meadows (upper right side).

**Figure 3.**
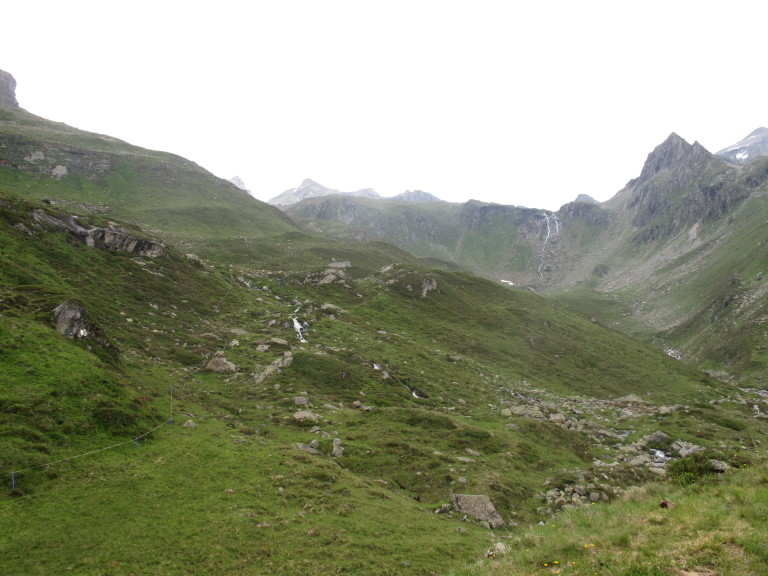
Alpine meadows and pastures are the predominant habitats in the study area.

**Figure 4.**
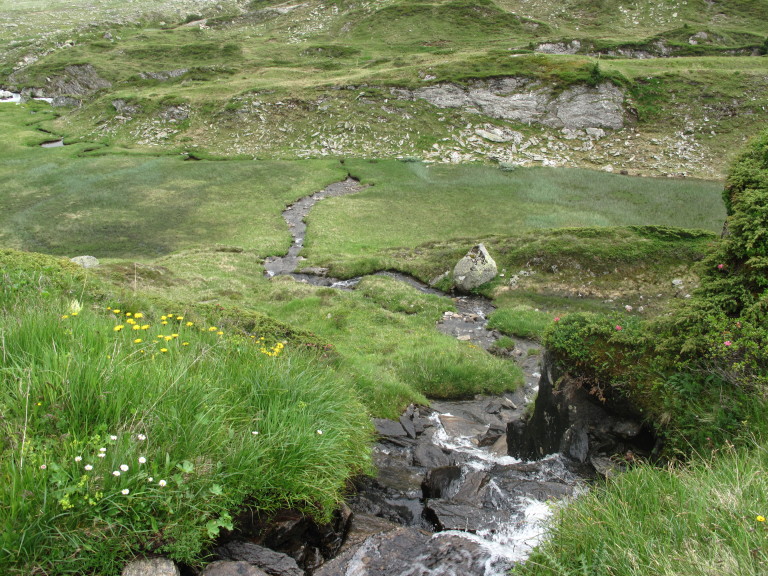
Wet meadows are present in the study area but are rarely frequented by wild bees.

The Zemmgrund is a valley located in the Zillertal Alps in Tyrol (Fig. 1) and is part of the Nature Park Zillertal Alps. It is located close to the main ridge of the Alps in northern direction and the southern boundary of the Upper Zemmgrund is the present-day border to Italy. Characteristic feature of the area are three glaciers, which are rapidly retreating at present (Gereben-Krenn et al. 2011): the Waxeggkees, Hornkees and the Schwarzensteinkees. The glaciers greatly influenced the geomorphology of the area and their moraines and remaining waters caused mosaics of diverse small-scaled habitats (Fig. 2). The predominant habitats of the area are alpine meadows and alpine pastures, especially above the treeline (Fig. 3). Other habitats are Swiss pine forests (*Pinus cembra* L.), aggregations of mountain pines (*Pinus mugo* Turra), tall forb meadows, dwarf shrub communities and wet meadows (Fig. 4). Great comprehensive information of the area, concerning the history, anthropogenic usage, climate and geology are available in Luzian and Pindur (2007). In addition, this compendium contains an extensive study about the flora of the area by Niklfeld and Schratt-Ehrendorfer (2007), a very useful source for melittologists!

## Checklists

### Checklist of the Apoidea of the Upper Zemmgrund area

#### Colletidae

#### Hylaeinae

#### Hylaeus nivalis

(Morawitz, 1867)

##### Materials

- **Type status:** Other material. **Occurrence:** occurrenceRemarks: on *Geum montanum* L.; recordedBy: S. Bossert; individualCount: 1; sex: male; **Location:** country: Austria; stateProvince: Tyrol; verbatimLocality: Zemmgrund; verbatimElevation: 2397; decimalLatitude: 47.036944; decimalLongitude: 11.829067; **Event:** samplingProtocol: manual catch; eventDate: 7-7-12; habitat: alpine meadow
- **Type status:** Other material. **Occurrence:** occurrenceRemarks: sheltering in *Leontodon hispidus* L.; recordedBy: S. Bossert; individualCount: 1; sex: male; **Location:** country: Austria; stateProvince: Tyrol; verbatimLocality: Zemmgrund; verbatimElevation: 2195; decimalLatitude: 47.031153; decimalLongitude: 11.821665; **Event:** samplingProtocol: manual catch; eventDate: 8-7-12; habitat: alpine meadow
- **Type status:** Other material. **Occurrence:** occurrenceRemarks: sheltering in *Leontodon hispidus* L.; recordedBy: S. Bossert; individualCount: 1; sex: male; **Location:** country: Austria; stateProvince: Tyrol; verbatimLocality: Zemmgrund; verbatimElevation: 2311 m; decimalLatitude: 47.03458; decimalLongitude: 11.82542; **Event:** samplingProtocol: manual catch; eventDate: 6-8-13; habitat: alpine meadow
- **Type status:** Other material. **Occurrence:** occurrenceRemarks: sheltering in *Leontodon helveticus* Mérat; recordedBy: S. Bossert; individualCount: 1; sex: male; **Location:** country: Austria; stateProvince: Tyrol; verbatimLocality: Zemmgrund; verbatimElevation: 2159 m; decimalLatitude: 47.028735; decimalLongitude: 11.818161; **Event:** samplingProtocol: manual catch; eventDate: 6-8-13; habitat: wet meadow
- **Type status:** Other material. **Occurrence:** occurrenceRemarks: visiting *Campanula barbata* L., afterwards *Leontodon helveticus*; recordedBy: S. Bossert; individualCount: 1; sex: female; **Location:** country: Austria; stateProvince: Tyrol; verbatimLocality: Zemmgrund; verbatimElevation: 2159 m; decimalLatitude: 47.028735; decimalLongitude: 11.818161; **Event:** samplingProtocol: manual catch; eventDate: 6-8-13; habitat: wet meadow

##### Distribution

The species occurs in the western European Alps and is strictly restricted to high-lying habitats (Dathe 1980, Dathe 2000).

#### Andrenidae

#### Andreninae

#### Andrena lapponica

Zetterstedt, 1838

##### Materials

- **Type status:** Other material. **Occurrence:** recordedBy: S. Bossert; individualCount: 1; sex: female; **Location:** country: Austria; stateProvince: Tyrol; verbatimLocality: Zemmgrund; verbatimElevation: 2115 m; decimalLatitude: 47.028050; decimalLongitude: 11.823114; **Event:** samplingProtocol: manual catch; eventDate: 4-7-12; habitat: alpine meadow / dwarf shrub community
- **Type status:** Other material. **Occurrence:** recordedBy: S. Bossert; individualCount: 1; sex: female; **Location:** country: Austria; stateProvince: Tyrol; verbatimLocality: Zemmgrund; verbatimElevation: 2125 m; decimalLatitude: 47.030206; decimalLongitude: 11.822817; **Event:** samplingProtocol: manual catch; eventDate: 4-7-12; habitat: alpine meadow / dwarf shrub community
- **Type status:** Other material. **Occurrence:** recordedBy: S. Bossert; individualCount: 1; sex: female; **Location:** country: Austria; stateProvince: Tyrol; verbatimLocality: Zemmgrund; verbatimElevation: 2010 m; decimalLatitude: 47.018500; decimalLongitude: 11.818060; **Event:** samplingProtocol: manual catch; eventDate: 6-7-12; habitat: alpine meadow
- **Type status:** Other material. **Occurrence:** recordedBy: S. Bossert; individualCount: 1; sex: female; **Location:** country: Austria; stateProvince: Tyrol; verbatimLocality: Zemmgrund; verbatimElevation: 2397m; decimalLatitude: 47.036944; decimalLongitude: 11.829067; **Event:** samplingProtocol: manual catch; eventDate: 7-7-12; habitat: alpine meadow
- **Type status:** Other material. **Occurrence:** recordedBy: S. Bossert; individualCount: 1; sex: female; **Location:** country: Austria; stateProvince: Tyrol; verbatimLocality: Zemmgrund; verbatimElevation: 2044m; decimalLatitude: 47.019165; decimalLongitude: 11.801332; **Event:** samplingProtocol: manual catch; eventDate: 4-7-13; habitat: alpine meadow

##### Distribution

A species with boreal-alpine distribution (Gusenleitner 1985).

##### Notes

The species is oligolectic on Ericaceae (Gusenleitner et al. 2012).

#### Andrena rogenhoferi

Morawitz, 1872

##### Materials

- **Type status:** Other material. **Occurrence:** recordedBy: S. Bossert; individualCount: 1; sex: 1 female; **Location:** country: Austria; stateProvince: Tyrol; verbatimLocality: Zemmgrund; verbatimElevation: 1904 m; decimalLatitude: 47.025414; decimalLongitude: 11.802853; **Event:** samplingProtocol: manual catch; eventDate: 5.7.12; habitat: Swiss pine forest / tall forb meadow

##### Distribution

According to Zettel et al. (2008), *Andrena rogenhoferi* is a high-alpine species distributed all over the European Alps.

##### Notes

Only few records of *Andrena rogenhoferi* have been reported so far (Zettel et al. 2008).

#### Andrena coitana

(Kirby, 1802)

##### Materials

- **Type status:** Other material. **Occurrence:** occurrenceRemarks: on *Leontodon hispidus*; recordedBy: S. Bossert; individualCount: 1; sex: male; **Location:** country: Austria; stateProvince: Tyrol; verbatimLocality: Zemmgrund; verbatimElevation: 2033 m; decimalLatitude: 47.023599; decimalLongitude: 11.813907; **Event:** samplingProtocol: manual catch; eventDate: 8-7-13; habitat: aggregation of mountain pines / tall forb meadow

##### Distribution

The species is distributed in great parts of Europe and Asia (Warncke 1981).

#### Andrena ruficrus

Nylander, 1848

##### Materials

- **Type status:** Other material. **Occurrence:** occurrenceRemarks: on *Geum montanum*; recordedBy: S. Bossert; individualCount: 1; sex: female; **Location:** country: Austria; stateProvince: Tyrol; verbatimLocality: Zemmgrund; verbatimElevation: 2001 m; decimalLatitude: 47.022145; decimalLongitude: 11.814224; **Event:** samplingProtocol: manual catch; eventDate: 7-7-13; habitat: alpine meadow

##### Distribution

According to Warncke (1981), the species is distributed between 43° and 70° north latitude in Europe and probably reaches Asia.

##### Notes

*Andrena ruficrus* is a rare species and Gusenleitner (1985) solely reports one single record of the species in Northern Tyrol.

#### Panurginae

#### Panurginus montanus

Giraud, 1861

##### Materials

- **Type status:** Other material. **Occurrence:** occurrenceRemarks: on *Potentilla* sp.; recordedBy: S. Bossert; individualCount: 1; sex: 1 male; **Location:** country: Austria; stateProvince: Tyrol; locality: Zemmgrund; verbatimElevation: 2117 m; decimalLatitude: 47.028291; decimalLongitude: 11.822605; **Event:** samplingProtocol: manual catch; eventDate: 07-04-12; habitat: alpine meadow / dwarf shrub community
- **Type status:** Other material. **Occurrence:** occurrenceRemarks: on yellow flowering Cichorioideae; recordedBy: S. Bossert; individualCount: 1; sex: 1 male; **Location:** country: Austria; stateProvince: Tyrol; locality: Zemmgrund; verbatimElevation: 1904 m; decimalLatitude: 47.025414; decimalLongitude: 11.802853; **Event:** samplingProtocol: manual catch; eventDate: 07-05-12; habitat: Swiss pine forest / tall forb meadow
- **Type status:** Other material. **Occurrence:** occurrenceRemarks: on *Leontodon hispidus*; recordedBy: S. Bossert; individualCount: 1; sex: 1 male; **Location:** country: Austria; stateProvince: Tyrol; locality: Zemmgrund; verbatimElevation: 2028 m; decimalLatitude: 47.023582; decimalLongitude: 11.813453; **Event:** samplingProtocol: manual catch; eventDate: 07-09-12; habitat: aggregation of mountain pines / tall forb meadow
- **Type status:** Other material. **Occurrence:** occurrenceRemarks: on *Potentilla* sp.; recordedBy: S. Bossert; individualCount: 2; sex: 2 males; **Location:** country: Austria; stateProvince: Tyrol; locality: Zemmgrund; verbatimElevation: 1882 m; decimalLatitude: 47.025294; decimalLongitude: 11.802437; **Event:** samplingProtocol: manual catch; eventDate: 07-03-13; habitat: Swiss pine forest / tall forb meadow

##### Distribution

The species is strictly restricted to the European Alps (Patiny 2003).

##### Notes

The males of *Panurginus montanus* can easily be determined with the key from Amiet et al. (2010).

#### Panurginus cf. montanus

Giraud, 1861

##### Materials

- **Type status:** Other material. **Occurrence:** occurrenceRemarks: on *Potentilla* sp.; recordedBy: S. Bossert; individualCount: 1; sex: 1 female; **Location:** country: Austria; stateProvince: Tyrol; locality: Zemmgrund; verbatimElevation: 2120 m; decimalLatitude: 47.028004; decimalLongitude: 11.822377; **Event:** samplingProtocol: manual catch; eventDate: 07-08-12; habitat: alpine meadow / dwarf shrub community
- **Type status:** Other material. **Occurrence:** occurrenceRemarks: on *Leontodon hispidus*; recordedBy: S. Bossert; individualCount: 2; sex: 2 females; **Location:** country: Austria; stateProvince: Tyrol; locality: Zemmgrund; verbatimElevation: 2028 m; decimalLatitude: 47.023582; decimalLongitude: 11.813453; **Event:** samplingProtocol: manual catch; eventDate: 07-09-12; habitat: aggregation of mountain pines / tall forb meadow
- **Type status:** Other material. **Occurrence:** occurrenceRemarks: on *Potentilla* sp.; recordedBy: S. Bossert; individualCount: 1; sex: 1 female; **Location:** country: Austria; stateProvince: Tyrol; locality: Zemmgrund; verbatimElevation: 2067 m; decimalLatitude: 47.025407; decimalLongitude: 11.815162; **Event:** samplingProtocol: manual catch; eventDate: 07-05-13; habitat: alpine meadow

##### Notes

The “cf.” status of the females is discussed below.

#### Halictidae

#### Halictinae

#### Lasioglossum albipes

(Fabricius, 1781)

##### Materials

- **Type status:** Other material. **Occurrence:** occurrenceRemarks: on *Leontodon* sp.; recordedBy: S. Bossert; individualCount: 1; sex: 1 female; **Location:** country: Austria; stateProvince: Tyrol; locality: Zemmgrund; verbatimElevation: 2397 m; decimalLatitude: 47.036944; decimalLongitude: 11.829067; **Event:** samplingProtocol: manual catch; eventDate: 07-07-12; habitat: alpine meadow

##### Distribution

The species is distributed throughout the whole Palaearctic (Ebmer 1988).

#### Lasioglossum alpigenum

(Dalla Torre, 1877)

##### Materials

- **Type status:** Other material. **Occurrence:** occurrenceRemarks: on *Leontodon hispidus*; recordedBy: S. Bossert; individualCount: 1; sex: 1 female; **Location:** country: Austria; stateProvince: Tyrol; locality: Zemmgrund; verbatimElevation: 2064 m; decimalLatitude: 47.025140; decimalLongitude: 11.814797; **Event:** samplingProtocol: manual catch; eventDate: 07-05-12; habitat: alpine meadow
- **Type status:** Other material. **Occurrence:** occurrenceRemarks: on *Potentilla* sp.; recordedBy: S. Bossert; individualCount: 1; sex: 1 female; **Location:** country: Austria; stateProvince: Tyrol; locality: Zemmgrund; verbatimElevation: 2001 m; decimalLatitude: 47.022312; decimalLongitude: 11.814189; **Event:** samplingProtocol: manual catch; eventDate: 07-06-12; habitat: alpine meadow

##### Distribution

*Lasioglossum alpigenum* is an alpine species with the main distribution in the European Alps (Ebmer 1988).

#### Lasioglossum fratellum

(Pérez, 1903)

##### Materials

- **Type status:** Other material. **Occurrence:** occurrenceRemarks: on *Leontodon* sp.; recordedBy: S. Bossert; individualCount: 1; sex: 1 female; **Location:** country: Austria; stateProvince: Tyrol; locality: Zemmgrund; verbatimElevation: 2004 m; decimalLatitude: 47.022352; decimalLongitude: 11.814313; **Event:** samplingProtocol: manual catch; eventDate: 07-09-12; habitat: alpine meadow
- **Type status:** Other material. **Occurrence:** occurrenceRemarks: on *Potentilla* sp. and *Myosotis* sp.; recordedBy: S. Bossert; individualCount: 2; sex: 2 females; **Location:** country: Austria; stateProvince: Tyrol; locality: Zemmgrund; verbatimElevation: 2001 m; decimalLatitude: 47.022312; decimalLongitude: 11.814189; **Event:** samplingProtocol: manual catch; eventDate: 07-06-12; habitat: alpine meadow

##### Distribution

Western Palaeartic (Ebmer 1988).

#### Lasioglossum morio

(Fabricius, 1793)

##### Materials

- **Type status:** Other material. **Occurrence:** occurrenceRemarks: on *Gentiana acaulis* L.; recordedBy: S. Bossert; individualCount: 1; sex: 1 female; **Location:** country: Austria; stateProvince: Tyrol; locality: Zemmgrund; verbatimElevation: 2120 m; decimalLatitude: 47.028004; decimalLongitude: 11.822377; **Event:** samplingProtocol: manual catch; eventDate: 07-08-12; habitat: alpine meadow
- **Type status:** Other material. **Occurrence:** occurrenceRemarks: on *Leontodon hispidus*; recordedBy: S. Bossert; individualCount: 1; sex: 1 female; **Location:** country: Austria; stateProvince: Tyrol; locality: Zemmgrund; verbatimElevation: 2072 m; decimalLatitude: 47.025460; decimalLongitude: 11.815305; **Event:** samplingProtocol: manual catch; eventDate: 07-07-12; habitat: alpine meadow

##### Distribution

Western Palaearctic (Ebmer 1988).

#### Rophitinae

#### Dufourea alpina

Morawitz, 1865

##### Materials

- **Type status:** Other material. **Occurrence:** occurrenceRemarks: on *Leontodon hispidus*; recordedBy: S. Bossert; individualCount: 5; sex: 5 males; **Location:** country: Austria; stateProvince: Tyrol; locality: Zemmgrund; verbatimElevation: 2117 m; decimalLatitude: 47.028291; decimalLongitude: 11.822605; **Event:** samplingProtocol: manual catch; eventDate: 07-04-12; habitat: alpine meadow / tall forb meadow
- **Type status:** Other material. **Occurrence:** occurrenceRemarks: on *Leontodon* sp.; recordedBy: S. Bossert; individualCount: 2; sex: 2 males; **Location:** country: Austria; stateProvince: Tyrol; locality: Zemmgrund; verbatimElevation: 1980 m; decimalLatitude: 47.019336; decimalLongitude: 11.807515; **Event:** samplingProtocol: manual catch; eventDate: 07-05-12; habitat: alpine meadow
- **Type status:** Other material. **Occurrence:** occurrenceRemarks: on *Phyteuma* sp.; recordedBy: S. Bossert; individualCount: 1; sex: 1 female; **Location:** country: Austria; stateProvince: Tyrol; locality: Zemmgrund; verbatimElevation: 2120 m; decimalLatitude: 47.028004; decimalLongitude: 11.822377; **Event:** samplingProtocol: manual catch; eventDate: 07-08-12; habitat: alpine meadow / dwarf shrub community
- **Type status:** Other material. **Occurrence:** occurrenceRemarks: on *Leontodon* sp.; recordedBy: S. Bossert; individualCount: 2; sex: 2 females; **Location:** country: Austria; stateProvince: Tyrol; locality: Zemmgrund; verbatimElevation: 2004 m; decimalLatitude: 47.022352; decimalLongitude: 11.814313; **Event:** samplingProtocol: manual catch; eventDate: 07-09-12; habitat: alpine meadow
- **Type status:** Other material. **Occurrence:** occurrenceRemarks: on yellow flowering Cichorioideae; recordedBy: S. Bossert; individualCount: 1; sex: 1 male; **Location:** country: Austria; stateProvince: Tyrol; locality: Zemmgrund; verbatimElevation: 2381 m; decimalLatitude: 47.036318; decimalLongitude: 11.828558; **Event:** samplingProtocol: manual catch; eventDate: 07-10-12; habitat: alpine meadow

##### Distribution

*Dufourea alpina* occurs in the Pyrenees and in the European Alps. Further it has been reported from the Balkan Peninsula (Ebmer 1988).

##### Notes

Together with *Panurginus montanus*, *Dufourea alpina* was probably the most common solitary bee species during the investigation period. Especially the males can easily be recognized since they often take shelter in flowers as mentioned in Amiet and Krebs (2012).

#### Dufourea paradoxa

Morawitz, 1867

##### Materials

- **Type status:** Other material. **Occurrence:** occurrenceRemarks: on *Leontodon hispidus*; recordedBy: S. Bossert; individualCount: 1; sex: 1 female; **Location:** country: Austria; stateProvince: Tyrol; locality: Zemmgrund; verbatimElevation: 2117 m; decimalLatitude: 47.028291; decimalLongitude: 11.822605; **Event:** samplingProtocol: manual catch; eventDate: 07-04-12; habitat: alpine meadow

##### Distribution

The specis has an altimontane distribution in the western and central Palaearctic (Ebmer 1988).

#### Megachilidae

#### Osmia inermis

(Zetterstedt, 1838)

##### Materials

- **Type status:** Other material. **Occurrence:** recordedBy: B. A. Gereben-Krenn; individualCount: 2; sex: 2 females; **Location:** country: Austria; stateProvince: Tyrol; locality: Zemmgrund; verbatimElevation: 2040 m; **Event:** eventDate: 07-06-12

##### Distribution

*Osmia inermis* was reported to be a boreal-alpine species, distributed throughout the Holarctic (Hicks 2009).

#### Osmia villosa

(Schenck, 1853)

##### Materials

- **Type status:** Other material. **Occurrence:** occurrenceRemarks: collecting petalum of *Cerastium alpinum* L.; recordedBy: J. F. Gokcezade; individualCount: 1; sex: 1 female; **Location:** country: Austria; stateProvince: Tyrol; locality: Zemmgrund; verbatimElevation: 1969 m; decimalLatitude: 47.021373; decimalLongitude: 11.811105; **Event:** samplingProtocol: manual catch; eventDate: 07-08-12; habitat: alpine meadow
- **Type status:** Other material. **Occurrence:** occurrenceRemarks: on *Leontodon* sp.; recordedBy: J. F. Gokcezade; individualCount: 1; sex: 1 female; **Location:** country: Austria; stateProvince: Tyrol; locality: Zemmgrund; verbatimElevation: 1985 m; decimalLatitude: 47.019727; decimalLongitude: 11.808610; **Event:** samplingProtocol: manual catch; eventDate: 07-08-12; habitat: alpine meadow
- **Type status:** Other material. **Occurrence:** recordedBy: J. F. Gokcezade; individualCount: 1; sex: 1 female; **Location:** country: Austria; stateProvince: Tyrol; locality: Zemmgrund; verbatimElevation: 2043 m; decimalLatitude: 47.024478; decimalLongitude: 11.812889; **Event:** samplingProtocol: manual catch; eventDate: 07-14-12; habitat: alpine meadow

##### Distribution

Central Europe (Warncke 1981). According to Gusenleitner et al. (2012), the species is distributed in high-lying habitats.

#### Apidae

#### Nomadinae

#### Nomada panzeri

Lepeletier, 1841

##### Materials

- **Type status:** Other material. **Occurrence:** occurrenceRemarks: sitting on a branch between flowers of *Rhododendron ferrugineum* L.; recordedBy: S. Bossert; individualCount: 1; sex: 1 female; **Location:** country: Austria; stateProvince: Tyrol; locality: Zemmgrund; verbatimElevation: 2120 m; decimalLatitude: 47.028004; decimalLongitude: 11.822377; **Event:** samplingProtocol: manual catch; eventDate: 07-08-12; habitat: alpine meadow / dwarf shrub community
- **Type status:** Other material. **Occurrence:** occurrenceRemarks: hovering over the ground and probably searching for a nest; recordedBy: S. Bossert; individualCount: 1; sex: 1 female; **Location:** country: Austria; stateProvince: Tyrol; locality: Zemmgrund; verbatimElevation: 1675 m; decimalLatitude: 47.032230; decimalLongitude: 11.778400; **Event:** samplingProtocol: manual catch; eventDate: 07-08-13; habitat: alpine pasture

##### Distribution

Northern, western and central Europe (Scheuchl 1995).

##### Notes

**Figure 5.**
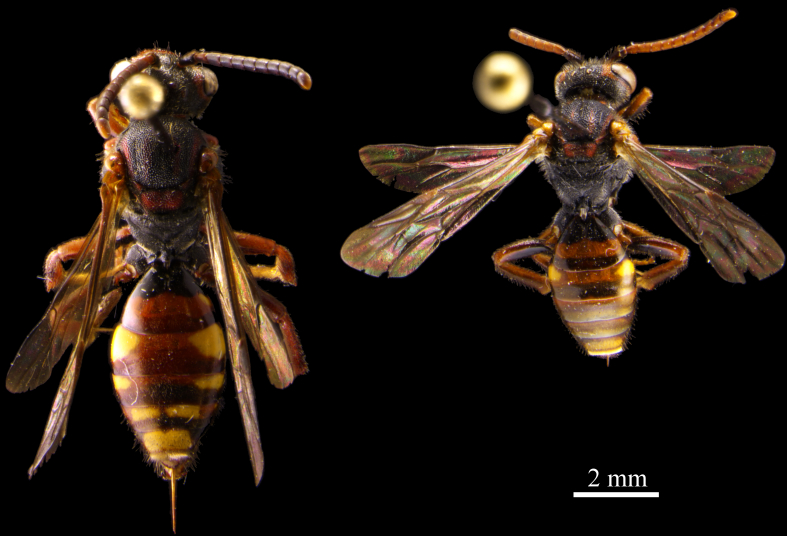
The two specimens of *Nomada panzeri* collected during the study. Note the great variation in size and color.

Following host species are mentioned in Scheuchl (1995): *Andrena fucata*, *Andrena helvola*, *Andrena lapponica* and *Andrena synadelpha*. The species is extremely variable in size and color (Fig. 5).

#### Apinae

#### Bombus bohemicus

Seidl, 1838

##### Materials

- **Type status:** Other material. **Occurrence:** occurrenceRemarks: on yellow flowering Cichorioideae; recordedBy: S. Bossert; individualCount: 1; sex: 1 queen; **Location:** country: Austria; stateProvince: Tyrol; locality: Zemmgrund; verbatimElevation: 1904 m; decimalLatitude: 47.024517; decimalLongitude: 11.802833; **Event:** samplingProtocol: manual catch; eventDate: 07-05-12; habitat: Swiss pine forest / tall forb meadow

##### Distribution

*Bombus bohemicus* has an Euro-Siberian distribution (Amiet 1996).

##### Notes

According to Amiet (1996), *Bombus lucorum* is the host species of *Bombus bohemicus*. It is presently not known if the other closely related species of the so-called *Bombus lucorum*-complex serve as host species as well.

#### Bombus cryptarum

(Fabricius, 1775)

##### Materials

- **Type status:** Other material. **Occurrence:** occurrenceRemarks: on *Rhododendron ferrugineum* L.; recordedBy: S. Bossert; individualCount: 1; sex: 1 queen; **Location:** country: Austria; stateProvince: Tyrol; locality: Zemmgrund; verbatimElevation: 2006 m; decimalLatitude: 47.022678; decimalLongitude: 11.813367; **Event:** samplingProtocol: manual catch; eventDate: 07-05-13; habitat: alpine meadow / tall forb meadow

##### Distribution

The species seems to have a boreal distribution in great parts of the Palaearctic and even reaches western North America (Williams et al. 2012).

##### Notes

The specimen belongs to a cryptic species complex consisting of *Bombus cryptarum*, *Bombus lucorum* and *Bombus magnus* but could positively be determined as *Bombus cryptarum* with the analyses of the nucleotide sequence of the COI gene. For details, see the discussion.

#### Bombus gerstaeckeri

Morawitz, 1882

##### Materials

- **Type status:** Other material. **Occurrence:** occurrenceRemarks: on *Aconitum napellus* L.; recordedBy: S. Bossert; individualCount: 3; sex: 3 females; **Location:** country: Austria; stateProvince: Tyrol; locality: Zemmgrund; verbatimElevation: 1603 m; decimalLatitude: 47.032362; decimalLongitude: 11.776317; **Event:** samplingProtocol: manual catch; eventDate: 08-08-13; habitat: tall forb meadow

##### Distribution

The species occurs in the Pyrenees, European Alps and on the Balkan Peninsula (Amiet 1996). Further it has been mentioned for the Carpathian and Caucasus Mountains (Ponchau et al. 2006).

##### Notes

*Bombus gerstaeckeri* is an oligolectic species and feeds on *Aconitum* spp. (Pittioni 1937, Amiet 1996, Utelli and Roy 2000).

#### Bombus hortorum

(L., 1761)

##### Materials

- **Type status:** Other material. **Occurrence:** occurrenceRemarks: on *Campanula barbata* L.; recordedBy: S. Bossert; individualCount: 1; sex: 1 female; **Location:** country: Austria; stateProvince: Tyrol; locality: Zemmgrund; verbatimElevation: 2057 m; decimalLatitude: 47.025236; decimalLongitude: 11.812656; **Event:** samplingProtocol: manual catch; eventDate: 07-10-12; habitat: aggregation of mountain pines / alpine meadow

##### Distribution

Palaearctic (Williams 1998, Williams 2014).

#### Bombus mendax

Gerstaecker, 1869

##### Materials

- **Type status:** Other material. **Occurrence:** recordedBy: S. Bossert; individualCount: 1; sex: 1 queen; **Location:** country: Austria; stateProvince: Tyrol; locality: Zemmgrund; verbatimElevation: 2079 m; decimalLatitude: 47.0256794; decimalLongitude: 11.8167508; **Event:** samplingProtocol: manual catch; eventDate: 07-06-13; habitat: alpine meadow

##### Distribution

Palaearctic (Williams 1998, Williams 2014). Amiet (1996) reports *Bombus mendax* to occur above 1500 m a.s.l. and Neumayer (1998) proposes the species to exceed even 3000 m a.s.l.

#### Bombus monticola

Smith, 1849

##### Materials

- **Type status:** Other material. **Occurrence:** occurrenceRemarks: on *Rhinanthus glacialis* Personnat; recordedBy: S. Bossert; individualCount: 1; sex: 1 female; **Location:** country: Austria; stateProvince: Tyrol; locality: Zemmgrund; verbatimElevation: 2041 m; decimalLatitude: 47.024797; decimalLongitude: 11.813171; **Event:** samplingProtocol: manual catch; eventDate: 07-10-12; habitat: alpine meadow

##### Distribution

Palaearctic (Williams 1998, Williams 2014).

#### Bombus pratorum

(L., 1761)

##### Materials

- **Type status:** Other material. **Occurrence:** occurrenceRemarks: on *Campanula barbata* L.; recordedBy: S. Bossert; individualCount: 1; sex: 1 female; **Location:** country: Austria; stateProvince: Tyrol; locality: Zemmgrund; verbatimElevation: 2041 m; decimalLatitude: 47.024797; decimalLongitude: 11.813171; **Event:** samplingProtocol: manual catch; eventDate: 07-10-12; habitat: alpine meadow

##### Distribution

Palaearctic (Williams 1998, Williams 2014).

#### Bombus pyrenaeus

Pérez, 1879

##### Materials

- **Type status:** Other material. **Occurrence:** occurrenceRemarks: on *Campanula* sp.; recordedBy: S. Bossert; individualCount: 1; sex: 1 female; **Location:** country: Austria; stateProvince: Tyrol; locality: Zemmgrund; verbatimElevation: 1979 m; decimalLatitude: 47.024259; decimalLongitude: 11.808473; **Event:** samplingProtocol: manual catch; eventDate: 07-05-12; habitat: alpine meadow
- **Type status:** Other material. **Occurrence:** occurrenceRemarks: on *Campanula barbata* L.; recordedBy: S. Bossert; individualCount: 1; sex: 1 female; **Location:** country: Austria; stateProvince: Tyrol; locality: Zemmgrund; verbatimElevation: 1896 m; decimalLatitude: 47.022036; decimalLongitude: 11.802090; **Event:** samplingProtocol: manual catch; eventDate: 07-05-12; habitat: alpine meadow
- **Type status:** Other material. **Occurrence:** occurrenceRemarks: on *Campanula barbata* L.; recordedBy: S. Bossert; individualCount: 1; sex: 1 female; **Location:** country: Austria; stateProvince: Tyrol; locality: Zemmgrund; verbatimElevation: 1896 m; decimalLatitude: 47.022036; decimalLongitude: 11.802090; **Event:** samplingProtocol: manual catch; eventDate: 07-05-12; habitat: alpine meadow
- **Type status:** Other material. **Occurrence:** occurrenceRemarks: on *Campanula* sp.; recordedBy: S. Bossert; individualCount: 1; sex: 1 female; **Location:** country: Austria; stateProvince: Tyrol; locality: Zemmgrund; verbatimElevation: 2004 m; decimalLatitude: 47.022304; decimalLongitude: 11.814452; **Event:** samplingProtocol: manual catch; eventDate: 07-06-12; habitat: alpine meadow
- **Type status:** Other material. **Occurrence:** recordedBy: S. Bossert; individualCount: 1; sex: 1 female; **Location:** country: Austria; stateProvince: Tyrol; locality: Zemmgrund; verbatimElevation: 2041 m; decimalLatitude: 47.024797; decimalLongitude: 11.813187; **Event:** samplingProtocol: manual catch; eventDate: 07-07-12; habitat: alpine meadow

##### Distribution

Palaearctic (Williams 1998, Williams 2014).

#### Bombus wurflenii

Radoszkowski, 1859

##### Materials

- **Type status:** Other material. **Occurrence:** occurrenceRemarks: on *Rhinanthus glacialis* Personnat; recordedBy: S. Bossert; individualCount: 1; sex: 1 female; **Location:** country: Austria; stateProvince: Tyrol; locality: Zemmgrund; verbatimElevation: 1972 m; decimalLatitude: 47.021912; decimalLongitude: 11.812132; **Event:** samplingProtocol: manual catch; eventDate: 07-05-12; habitat: alpine meadow

##### Distribution

Palaearctic (Williams 1998, Williams 2014).

## Analysis

In total, 61 specimens were collected, representing 24 species from 8 genera. The list of bumblebee species provided in Penninger (2008) can be complemented with 4 species. The note about *Bombus lucorum* from this source is not evaluated due to the current unreliability of morphological identification of the species. Combining these records, 30 bee species have been recorded for the area. Of these, 15 are representatives of the genus *Bombus*.

## Discussion

With increasing altitude, the climatic conditions in alpine environments become more extreme (Franz 1979). Especially the decreasing temperature (Fig. 1) and increasing insolation are of importance for terrestrial arthropods above the timberline (Sømme 1989). This also applies for bees: due to the short and cool summers in alpine regions in the European Alps, Neumayer and Paulus (1999) conclude a phenological window of solely three months for bumblebees to complete their life cycle. With respect to the nearly completed snowmelt in the study area by mid of June, it can be safely assumed that the three investigation periods were extensive enough to collect the majority of bee species that may occur in the area. Nonetheless, the species list above cannot be assumed to be complete with certainty. Therefore the investigation periods were not evenly distributed throughout the season since no collections have been conducted in September. Also the study area is almost completely restricted to the Upper Zemmgrund and species which potentially occur below 1900 m altitude are absent from the species list. This becomes particularly apparent when comparing the records with the species list of Kopf et al. (2010). This species list is based on collections from July 17-19, 2009 by four persons on eight collection sites approx. 13 to 17 km from the Zemmgrund Area as the crow flies. Therefore it is comparable by place, time and collection effort but differs in the altitudinal range: the collections were conducted between 1760 m and approx. 950 m a.s.l. Only seven of 47 species collected by Kopf et al. (2010) can be found in both lists, namely the widely distributed bumblebees *Bombus hortorum*, *Bombus monticola*, *Bombus pratorum*, *Bombus pyrenaeus* and the widespread *Lasioglossum albipes*, *Lasioglossum fratellum* and *Lasioglossum morio*. Therefore a great number of additional species can be expected at lower altitudes of the Zemmgrund area. Further the comparison of the lists indicates a decreasing species diversity along the rising altitudinal gradient. This is in line with the described species decrease of terrestrial arthropods at the timberline (Sømme 1989). However, the species composition in the Upper Zemmgrund clearly reflects the high altitudes of the study area, since the majority of species have at least a montane distribution. Several records belong to explicit high-mountain species, such as *Hylaeus nivalis* (Dathe 1980, Dathe 2000), *Andrena rogenhoferi* (Gusenleitner 1985, Zettel et al. 2008), *Panurginus montanus* (Patiny 2003), *Lasioglossum alpigenum* (Ebmer 1988), *Dufourea alpina* (Ebmer 1988) and the bumblebee species *Bombus mendax*, *Bombus monticola* and *Bombus wurflenii* (Neumayer 1998).

Some species determinations must be discussed: Since females from *Panurginus montanus* cannot be separated from females of the closely related *Panurginus sericatus* Warncke, 1972 with the key of Amiet et al. (2010), the collected females are marked with “cf”. The species status of *Panurginus sericatus* has been doubted (Ebmer 2001), but is valid after Patiny (2003). However, since the males can easily be assigned by the shape of the gonostylus and both sexes were observed in the same area, it seems likely that the females belong to *Panurginus montanus*.

**Figure 6.**
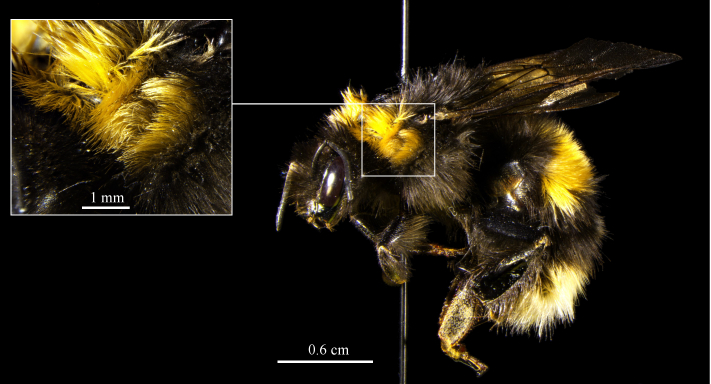
The voucher of the collected queen of *Bombus cryptarum*. The black “S”-shape in the first collar is clearly visible.

Another species with difficult determination is *Bombus cryptarum*. An identification based on the characteristic color patterns of queens was shown to be unreliable (Carolan et al. 2012), and an examination on the reliability of traits described in the common keys is urgently needed since several characters overlap. In contrast, sequence analyses of the COI gene represents a confident method for identification (e.g. Bertsch et al. 2005, Murray et al. 2008, Bertsch 2009, Carolan et al. 2012, Williams et al. 2012). A BLAST search of the obtained 609 bp long sequence from the collected specimen (Suppl. material 2, GenBank acc. no. KJ787691) revealed an identity of 99% with a query cover of 100% to a *Bombus cryptarum* voucher (GenBank acc. no. JQ843372.1) and the next 40 hits by total score were assigned to *Bombus cryptarum*. Therefore it can safely be assumed that the specimen belongs to *Bombus cryptarum*. The specimen shows the ‘S’-shape of black hairs in the first collar (Fig. 6), which has been considered a characteristic trait for queens in the literature (Rasmont 1981, Rasmont 1984, Bertsch 1997, Bertsch et al. 2004). After Rasmont (pers. comm.), specimens showing the “S” belong to the subspecies *Bombus cryptarum cryptarum*. However, since Carolan et al. (2012) could show that this trait seems to be unreliable, further discussion about species identification of the cryptic species of the *Bombus lucorum* complex based on color patterns cannot be conducted until more safely determined specimens are accessible.

As with many species of the genus, *Nomada panzeri* shows a great variation in color and size (Schwarz 1986). This also applies to the two collected specimens from this study which vary considerably (Fig. 5). The specimens were determined and labeled by the European expert for this group, Maximilian Schwarz, as “*Nomada glabella* auct.” (*Nomada glabella* Thomson, 1870) which is a junior synonym to *Nomada panzeri* (Schwarz 1986). Burger (2005) disagrees with the synonymy and argues with the clear distinguishability described in Stoeckhert (1930). Further he proposes differences in distributional patterns between *Nomada panzer* and *Nomada glabella* and solely mentions *Andrena lapponica* and *Andrena fucata* as host species of *Nomada glabella*. In contrast, the majority of authors agree with the synonymy (e.g. Schwarz et al. 1996, Smit 2004, Straka et al. 2007, Westrich et al. 2008, Nilsson 2010, Gusenleitner et al. 2012). However, it can safely be assumed that the host species of *Nomada panzer* in the study area is *Andrena lapponica*.

## Supplementary Material

Supplementary material 1 Plant species and flower visiting bee species Data type: Flower records
Brief description: The list provides the plant species on which flower visits by bees could be observed during this study. Additionally, the respective flower visiting bee species are listed.
File: oo_8085.xlsx Silas Bossert

Supplementary material 2 Partial COI sequence of the Bombus cryptarum voucher Data type: mitochondrial DNA sequence
Brief description: The fasta file contains the partial cds of the COI gene from the *Bombus cryptarum* voucher investigated in this study.
File: oo_8076.fas Silas Bossert

XML Treatment for Colletidae

XML Treatment for Hylaeinae

XML Treatment for Hylaeus nivalis

XML Treatment for Andrenidae

XML Treatment for Andreninae

XML Treatment for Andrena lapponica

XML Treatment for Andrena rogenhoferi

XML Treatment for Andrena coitana

XML Treatment for Andrena ruficrus

XML Treatment for Panurginae

XML Treatment for Panurginus montanus

XML Treatment for Panurginus cf. montanus

XML Treatment for Halictidae

XML Treatment for Halictinae

XML Treatment for Lasioglossum albipes

XML Treatment for Lasioglossum alpigenum

XML Treatment for Lasioglossum fratellum

XML Treatment for Lasioglossum morio

XML Treatment for Rophitinae

XML Treatment for Dufourea alpina

XML Treatment for Dufourea paradoxa

XML Treatment for Megachilidae

XML Treatment for Osmia inermis

XML Treatment for Osmia villosa

XML Treatment for Apidae

XML Treatment for Nomadinae

XML Treatment for Nomada panzeri

XML Treatment for Apinae

XML Treatment for Bombus bohemicus

XML Treatment for Bombus cryptarum

XML Treatment for Bombus gerstaeckeri

XML Treatment for Bombus hortorum

XML Treatment for Bombus mendax

XML Treatment for Bombus monticola

XML Treatment for Bombus pratorum

XML Treatment for Bombus pyrenaeus

XML Treatment for Bombus wurflenii

## References

[B669269] Altschul Stephen F, Gish Warren, Miller Webb, Myers Eugene W, Lipman David J (1990). Basic Local Alignment Search Tool. J. Mol. Biol..

[B669280] Amiet F. (1996). HymenopteraApidae, 1. Teil - Allgemeiner Teil, Gattungsschlüssel, die Gattungen *Apis*, *Bombus* und *Psithyrus*.

[B669325] Amiet F., Krebs A. (2012). Bienen Mitteleuropas - Gattungen, Lebensweise, Beobachtung.

[B669334] Amiet F., Neumeyer R., Müller A. (1999). Fauna Helvetica. Apidae 2 - *Colletes*, *Dufourea*, *Hylaeus*, *Nomia*, *Nomioides*, *Rhophitoides*, *Rophites*, *Sphecodes*, *Systropha*.

[B669289] Amiet F., Hermann M., Müller A., Neumeyer R. (2010). Fauna Helvetica. Apidae 6 - *Andrena*, *Melitturga*, *Panurginus*.

[B669298] Amiet F., Herrmann M., Müller A., Neumeyer R. (2001). Fauna Helvetica. Apidae 3 - *Halictus*, *Lasioglossum*.

[B669307] Amiet F., Herrmann M., Müller A., Neumeyer R. (2004). Fauna Helvetica. Apidae 4 - *Anthidium*, *Chelostoma*, *Coelioxys*, *Dioxys*, *Heriades*, *Lithurgus*, *Megachile*, *Osmia*, *Stelis*.

[B669316] Amiet F., Herrmann M., Müller A., Neumeyer R. (2007). Fauna Helvetica. Apidae 5 - *Ammobates*, *Ammobatoides*, *Anthophora*, *Biastes*, *Ceratina*, *Dasypoda*, *Epeoloides*, *Epeolus*, *Eucera*, *Macropis*, *Melecta*, *Melitta*, *Nomada*, *Pasites*, *Tetralonia*, *Thyreus*, *Xylocopa*.

[B673737] Bellmann H., Hellrigl K., Hellrigl K. (1996). Apoidea (Mellifera)-Bienen oder Blumenwespen. Die Tierwelt Südtirols.

[B669343] Bertsch A. (1997). Abgrenzung der Hummel-Arten *Bombus
cryptarum* und *Bombus
lucorum* mittels männlicher Labialdrüsen-Sekrete und morphologischer Merkmale (Hymenoptera, Apidae). Entomol. Gener..

[B669353] Bertsch A. (2009). Barcoding cryptic bumblebee taxa: *Bombus
lucorum*, *Bombus
crytarum* and *Bombus
magnus*, a case study. Beiträge zur Entomologie.

[B669363] Bertsch A., Schweer H., Titze A. (2004). Discrimination of the bumblebee species *Bombus
lucorum*, *Bombus
cryptarum* and *Bombus
magnus* by morphological characters and male labial gland secretions. Beiträge zur Entomologie.

[B669373] Bertsch A., Schweer H., Titze A., Tanaka H. (2005). Male labial gland secretions and mitochondrial DNA markers support species status of *Bombus
cryptarum* and *Bombus
magnus* (Hymenoptera, Apidae). Insectes Sociaux.

[B673855] Burger F. (2005). Rote Liste Wildbienen - Materialien zu Naturschutz und Landschaftspflege.

[B669383] Carolan J. C., Murray T. E., Fitzpatrick Ú., Crossley J., Schmidt H., Cederberg B., McNally L., Paxton R. J., Williams P. H., Brown M. J.F. (2012). Colour Patterns Do Not Diagnose Species: Quantitative Evaluation of a DNA Barcoded Cryptic Bumblebee Complex. PloS one.

[B669399] Dalla Torre K. W. v. (1873). Beitrag zur Kenntnis der Hymenopterenfauna Tirols. - Die Apiden Tirols in ihrer horizontalen und vertikalen Verbreitung. Ztschr. Ferdinandeum Innsbruck.

[B669409] Dalla Torre K. W. v. (1877). Die Apiden Tirols. Fortsetzung und Schluss. Ztschr. Ferdinandeum Innsbruck.

[B669419] Dalla Torre K. W. v. (1877). Entomologische Alpenfauna. Ent. Nachr..

[B669429] Dalla Torre K. W. v. (1879). Bemerkungen zur Gattung Bombus Latr. I. 1. Die Bombus-Arten Tirols. Ber. nat.-med. Ver. Innsbruck.

[B669439] Dalla Torre K. W. v. (1882). Bemerkungen zur Gattung *Bombus* Latr. II. 3. Zur Synonymie und geographischen Verbreitung der Gattung Bombus Latr.. Ber. nat.-med. Ver. Innsbruck.

[B669449] Dathe H. H. (1977). Diagnosen zu den europäischen Arten der *Hylaeus
nivalis*-Gruppe (Hymenoptera: Apidae, Colletidae). Mitt. zool. Mus. Berlin.

[B669459] Dathe H. H. (1980). Die Arten der Gattung *Hylaeus* F. in Europa (Hymenoptera: Apoidea, Colletidae). Mitt. zool. Mus. Berlin.

[B673784] Dathe H. H. (2000). Studien zur Systematik und Taxonomie der Gattung *Hylaeus* F. (3). Revision der *Hylaeus
nivalis*-Gruppe in Europa und Klärung weiterer westpaläarktischer Arten (Apidae, Colletinae). Beitr. Ent..

[B669469] Ebmer A. W. (1969). Die Bienen des Genus *Halictus* Latr. sl. im Großraum von Linz (Hymenoptera, Apidae). Teil I. Nat. Jb. Linz.

[B669479] Ebmer A. W. (1971). Die Bienen des Genus *Halictus* Latr. s.l. im Großraum von Linz (Hymenoptera, Apidae) Teil III. Nat. Jb. Linz.

[B669489] Ebmer A. W. (1984). Die westpaläarktischen Arten der Gattung *Dufourea* Lepeletier 1841 mit illustrierten Bestimmungstabellen (Insecta: Hymenoptera: Apoidea: Halictidae: Dufoureinae). Senckenbergiana biol..

[B669499] Ebmer A. W. (1988). Kritische Liste der nicht-parasitischen Halictidae Österreichs mit Berücksichtigung aller mitteleuropäischen Arten (Insecta: Hymenoptera: Apoidea: Halictidae). Linzer biol. Beitr..

[B669509] Ebmer A. W. (2001). Hymenopterologische Notizen aus Österreich-14 (Insecta: Hymenoptera: Apoidea). Linzer biol. Beitr..

[B669519] Fischer M. A., Oswald K., Adler W. (2008). Exkursionsflora für Österreich, Liechtenstein und Südtirol.

[B669528] Folmer O., Black M., Hoeh W., Lutz R., Vrijenhoek R (1994). DNA primers for amplification of mitochondrial cytochrome c oxidase subunit I from diverse metazoan invertebrates. Molecular marine biology and biotechnology.

[B722654] Franz H (1979). Ökologie der Hochgebirge.

[B669539] Gereben-Krenn B. A., Krenn Harald W, Strodl Markus A (2011). Initial Colonization of New Terrain in an Alpine Glacier Foreland by Carabid Beetles (Carabidae, Coleoptera). Arctic, Antarctic, and Alpine Research.

[B669549] Gokcezade J. F., Gereben-Krenn B. A., Neumayer J., Krenn H. W. (2010). Feldbestimmungsschlüssel für die Hummeln Österreichs, Deutschlands und der Schweiz. Linzer biologische Beiträge.

[B669559] Gusenleitner Fritz (1985). Angaben zur Kenntnis der Bienengattung *Andrena* in Nordtirol (Österreich). Ber. nat.-med. Ver. Innsbruck.

[B671954] Gusenleitner F, Schwarz M, Mazzucco K, Schuster R. (2012). Apidae (Insecta: Hymenoptera). Checklisten der Fauna Österreichs, No. 6.

[B669569] Hall Tom A (1999). BioEdit: a user-friendly biological sequence alignment editor and analysis program for Windows 95/98/NT. Nucleic Acids Symposium Series.

[B669579] Hellrigl Klaus (2003). Faunistik der Ameisen und Wildbienen Südtirols (Hymenoptera: Formicidae et Apoidea). Gredleriana.

[B669589] Hellrigl Klaus (2006). Synopsis der Wildbienen Südtirols: (Hymenoptera: Apidae). Forest observer.

[B669599] Hellrigl Klaus, Franke Rolf (2004). Faunistik der Wildbienen Südtirols: 1. Nachtrag (Hymenoptera: Apoidea). Forest observer.

[B721530] Hicks Barry (2009). Observations of the nest structure of *Osmia
inermis* (Hymenoptera: Megachilidae) from Newfoundland, Canada. Journal of the Acadian Entomological Society.

[B669609] Hiebl J, Reisenhofer S, Auer I, Böhm R, Schöner W (2011). Multi-methodical realisation of Austrian climate maps for 1971–2000. Advances in Science and Research.

[B669620] Kopf T. (2008). Die Bienenfauna (Hymenoptera: Apidae) des Schlerngebietes (Südtirol, Italien) mit Angaben zu den Artengemeinschaften ausgewählter Lebensräume. Gredleriana.

[B673723] Kopf T., Zettel H, Link A., Ockermüller E., Pagitz K. (2010). Hautflügler - Pflanzenwespen und ausgewählte Stechimmen-Familien (Hymenoptera: "Symphyta" et Aculeata partim: Apidae, Chrysididae, Sapygidae, Sphecidae, Vespidae). GEO-Tag der Artenvielfalt 2009 in Tirol - Naturpark Zillertal. In: Wissenschaftliches Jahrbuch der Tiroler Landesmuseen.

[B669630] Kuhlmann M., Tumbrinck K. (1996). Wildbienen- und Wespenfunde (Hymenoptera Aculeata) aus dem Kleinwalsertal und aus den Silvretta-Alpen. Jb. Vorarlberger Landesmuseumsvereins-Freunde d. Landeskunde.

[B669640] Luzian Roland, Pindur Peter (2007). Prähistorische Lawinen: Nachweis und Analyse holozäner Lawinenereignisse in den Zillertaler Alpen, Österreich.

[B669649] Mauss V. (1994). Bestimmungsschlüssel für Hummeln. 6. Auflage DJN (Hrsg.).

[B721671] Michener C. D. (2007). The Bees of the World.

[B669659] Murray T. E., Fitzpatrick U., Brown M. J.F., Paxton R. J. (2008). Cryptic species diversity in a widespread bumble bee complex revealed using mitochondrial DNA RFLPs. Conservation Genetics.

[B721658] Neumayer J (1998). Habitatpräferenzen alpiner Hummelarten (Hymenoptera, Apidae, *Bombus*, *Psithyrus*): Meereshöhe und Lage im Gebirgsrelief als Faktoren der Nischentrennung.. Wissenschaftliche Mitteilungen Nationalpark Hohe Tauern.

[B669669] Neumayer J., Kofler A. (2005). Zur Hummelfauna des Bezirkes Lienz (Osttirol, Österreich)(Hymenoptera: Apidae, *Bombus*). Linzer biol. Beitr..

[B722683] Neumayer J, Paulus H. F. (1999). Ökologie alpiner Hummelgemeinschaften: Blütenbesuch, Ressourcenaufteilung und Energiehaushalt. Untersuchungen in den Ostalpen Österreichs. Stapfia.

[B673751] Niklfeld H., Schratt-Ehrendorfer L., Luzian R., Pindur P. (2007). Zur Flora des Zemmgrunds in den Zillertaler Alpen - Ein Auszug aus den Ergebnissen der Floristischen Kartierung Österreichs. Prähistorische Lawinen. Nachweis und Analyse holozäner Lawinenereignisse in den Zillertaler Alpen, Österreich.

[B669679] Nilsson L Anders (2010). The type material of Swedish bees (Hymenoptera, Apoidea) IV. Bees from Thomson’s collection. Entomologisk Tidskrift.

[B669689] Patiny Sébastien (2003). Contemporary distributions of *Panurginus* species and subspecies in Europe (Apoidea: Andrenidae: Panurginae). Proceedings of the 13th International Colloquium of the European Invertebrate Survey, Leiden, 2-5 September 2001.

[B673714] Penninger H (2008). Aktivität alpiner Hummeln in Abhängigkeit klimatischer Faktoren.

[B721572] Pittioni B. (1937). Bestäubung und Nektarraub beim Gelben Eisenhut (*Aconitum
vulparia* Rchb). Aus der Heimat, Stuttgart.

[B721592] Ponchau O., Iserbyt S., Verhaeghe J. -C., Rasmont P. (2006). Is the caste-ratio of the oligolectic bumblebee *Bombus
gerstaeckeri* Morawitz (Hymenoptera: Apidae) biased to queens?. Annales de la Société Entomologique de France.

[B673765] QGIS Development Team (2014). QGIS Geographic Information System. http://qgis.osgeo.org.

[B673917] Rasmont Pierre (1981). Contribution à l'étude des bourdons du genre *Bombus* Latreille, 1802 sensu stricto (*Hymenoptera*, *Apidæ, Bombinæ*).

[B669729] Rasmont P. (1984). Les bourdons du genre *Bombus* Latreille sensu stricto en Europe Occidentale et Centrale (Hymenoptera, Apidae). Spixiana.

[B669739] Rothmaler W. (2009). Exkursionsflora von Deutschland 3. Gefäßpflanzen: Atlasband. Herausgeg. v. E.J. Jäger & K. Werner. 11. Auflage.

[B669748] Sambrook Joseph, Fritsch Edward F, Maniatis Thomas (1989). Molecular cloning.

[B669757] Schedl W. (1982). Über aculeate Hautflügler der zentralen Ötztaler Alpen (Tirol, Österreich) (Insecta: Hymenoptera). Ber. nat.-med. Ver. Innsbruck.

[B669767] Scheuchl E. (1995). Illustrierte Bestimmungstabellen der Wildbienen Deutschlands und Österreichs. Band I: Anthophoridae.

[B669776] Scheuchl E. (2006). Illustrierte Bestimmungstabellen der Wildbienen Deutschlands und Österreichs. Band II: Megachilidae - Melittidae. Zweite erweiterte Auflage.

[B669785] Schletterer August (1887). Die Bienen Tirols. Jber. k. k. Staatsrealschule II. Bez. Wien.

[B669795] Schmid-Egger C. (2011). Die Stechimmenfauna (Hymenoptera Aculeata) im Naturpark Tiroler Lech in Österreich. Linzer biol. Beitr..

[B669805] Schmid-Egger C., Scheuchl E. (1997). Illustrierte Bestimmungstabellen der Wildbienen Deutschlands und Österreichs. Band III: Andrenidae.

[B669814] Schuler Klaus (1982). Blütenbesuch durch Insekten an *Solidago
canadensis* und *Solidago
virgaurea*, eine vergleichende Studie. Ber. nat.-med. Ver. Innsbruck.

[B669824] Schwarz M. (1986). Revision der Nomada-Arten der Sammlung C. G. Thomson (Hymenoptera, Apoidea). Entomofauna.

[B669834] Schwarz M., Gusenleitner F., Westrich P., Dathe H. H. (1996). Katalog der Bienen Österreichs, Deutschlands und der Schweiz (Hymenoptera, Apidae). Entomofauna.

[B669844] Smit Jan (2004). De Wespbijen (*Nomada*) van Nederland (Hymenoptera: Apidae). Nederlandse Faunistische Mededelingen.

[B722673] Sømme L (1989). Adaptions of Terrestrial Arthropods to the Alpine Environment. Biological Reviews.

[B669854] Stöckl Petra (1996). Artengarnitur und Blütenbesuch von Wildbienen an vier xerothermen Standorten zwischen Kranebitten und Zirl (Nordtirol, Österreich)(Hymenoptera: Apoidea). Ber. nat.-med. Ver. Innsbruck.

[B669864] Stöckl Petra (1998). Die Wildbienen ausgewählter Xerothermstandorte des Oberinntales (Nordtirol, Österreich). Ber. nat.-med. Ver. Innsbruck.

[B669874] Stöckl P. (2000). Synopsis der Megachilinae Nord-und Südtirols (Österreich, Italien). Ber. nat.-med. Ver. Innsbruck.

[B673864] Stoeckhert E., Schmiedeknecht Otto (1930). *Nomada* F.. Die Hymenopteren Nord- und Mitteleuropas.

[B673827] Straka J., Bogusch Petr, Přidal Antonín, Bogusch Petr, Straka Jakub, Kment P (2007). Apoidea: Apiformes (včely). Annotated checklist of the Aculeata (Hymenoptera) of the Czech Republic and Slovakia. Komentovaný seznam žahadlových blanokřídlých (Hymenoptera: Aculeata) České republiky a Slovenska.

[B721582] Utelli A. B., Roy B. A. (2000). Pollinator abundance and behavior on *Aconitum
lycoctonum* (Ranunculaceae): an analysis of the quantity and quality components of pollination. Oikos.

[B721510] Warncke K. (1981). Die Bienen des Klagenfurter Beckens (Hymenoptera, Apidae). Carinthia II.

[B721690] Waters J, Darvill B, Lye G. C., Goulson D (2011). Niche differentiation of a cryptic bumblebee complex in the Western Isles of Scotland. Insect Conservation and Diversity.

[B669884] Westrich Paul, Frommer Ulrich, Mandery Klaus, Riemann Helmut, Ruhnke Haike, Saure Christoph, Voith Johannes (2008). Rote Liste der Bienen Deutschlands (Hymenoptera, Apidae)(4. Fassung, Dezember 2007). Eucera.

[B721648] Williams P. H. (1998). An annotated checklist of bumble bees with an analysis of patterns of description (Hymenoptera: Apidae, Bombini). Bulletin of The Natural History Museum (Entomology).

[B721680] Williams P. H. (2000). Are *Bombus
lucorum* and *magnus* separate species?. BWARS Newsletter.

[B721639] Williams P. H. (2014). *Bombus* - Species world-wide listed by old and new subgenera. Accessed 09/06/2014. http://www.nhm.ac.uk/research-curation/research/projects/bombus/subgenericlist.html.

[B669897] Williams P. H., Brown M. J.F., Carolan J. C., An J., Goulson D., Aytekin A. M., Best L. R., Byvaltsev A. M., Cederberg B., Dawson R., Huang J., Ito M., Monfared A., Raina R. H., Schmid-Hempel P., Sheffield C. S., Šima P., Xie Z. (2012). Unveiling cryptic species of the bumblebee subgenus *Bombus* s. str. worldwide with COI barcodes (Hymenoptera: Apidae). Systematics and Biodiversity.

[B721489] Zettel H, Ebmer A. W., Wiesbauer H. (2008). Zur Kenntnis der Wildbienen (Hymenoptera: Apidae) in Wien, Niederösterreich und dem Burgen land (Österreich) – 4. Beiträge zur Entomofaunistik.

